# Regulation of Energy Substrate Metabolism in Endurance Exercise

**DOI:** 10.3390/ijerph18094963

**Published:** 2021-05-07

**Authors:** Abdullah F. Alghannam, Mazen M. Ghaith, Maha H. Alhussain

**Affiliations:** 1 Lifestyle and Health Research Center, Health Sciences Research Center, Princess Nourah bInt. Abdulrahman University, Riyadh 84428, Saudi Arabia; 2 Faculty of Applied Medical Sciences, Laboratory Medicine Department, Umm Al-Qura University, Al Abdeyah, Makkah 7607, Saudi Arabia; mmghaith@uqu.edu.sa; 3 Department of Food Science and Nutrition, College of Food and Agriculture Sciences, King Saud University, Riyadh 11451, Saudi Arabia; mhussien@ksu.edu.sa

**Keywords:** energy, metabolism, carbohydrate, protein, fat, endurance exercise

## Abstract

The human body requires energy to function. Adenosine triphosphate (ATP) is the cellular currency for energy-requiring processes including mechanical work (i.e., exercise). ATP used by the cells is ultimately derived from the catabolism of energy substrate molecules—carbohydrates, fat, and protein. In prolonged moderate to high-intensity exercise, there is a delicate interplay between carbohydrate and fat metabolism, and this bioenergetic process is tightly regulated by numerous physiological, nutritional, and environmental factors such as exercise intensity and duration, body mass and feeding state. Carbohydrate metabolism is of critical importance during prolonged endurance-type exercise, reflecting the physiological need to regulate glucose homeostasis, assuring optimal glycogen storage, proper muscle fuelling, and delaying the onset of fatigue. Fat metabolism represents a sustainable source of energy to meet energy demands and preserve the ‘limited’ carbohydrate stores. Coordinated neural, hormonal and circulatory events occur during prolonged endurance-type exercise, facilitating the delivery of fatty acids from adipose tissue to the working muscle for oxidation. However, with increasing exercise intensity, fat oxidation declines and is unable to supply ATP at the rate of the exercise demand. Protein is considered a subsidiary source of energy supporting carbohydrates and fat metabolism, contributing to approximately 10% of total ATP turnover during prolonged endurance-type exercise. In this review we present an overview of substrate metabolism during prolonged endurance-type exercise and the regulatory mechanisms involved in ATP turnover to meet the energetic demands of exercise.

## 1. Introduction

Humans are capable of performing extraordinary feats; with both natural endowment and sport-specific training, athletes can perform various activities with astonishing power, speed, skill and tolerance [1]. Even for non-athletes, the physical demands of locomotion, lifting, climbing and exercising pose a major bodily challenge. Before all these movements can occur, a source of energy must be obtained. This comes in the form of adenosine triphosphate (ATP): the cellular currency for all energy-requiring processes including mechanical work [2]. ATP turnover can increase 100-fold from rest to exercise [3,4] and thus poses a major energetic challenge [5]. Remarkably, skeletal muscles are able to match ATP supply with demand under many different physiological and environmental stimuli, highlighting the high metabolic plasticity [6,7,8] of this most abundant tissue in humans [9]. Given the limited amount of intracellular ATP (sustaining ≈2 s of maximal exercise), infers that other metabolic pathways to resynthesise ATP must be activated to sustain skeletal muscle work [10].

Research into exercise metabolism and the substrates necessary to support different activities has been extensive and spans just over a century [11,12,13,14,15,16,17,18,19,20,21,22,23,24,25]. Whether directly (daily feeding) or indirectly (endogenous stores), carbohydrates, fat and protein supply the energy requirements for the human body enabling the resynthesis of ATP [26,27]. Carbohydrates and fat are the primary substrates for energy metabolism in humans during prolonged endurance-type exercise [28,29]. Carbohydrates are efficient, costing 11% less oxygen during steady-state exercise [22]. The reliance on this substrate over fats increases exponentially with exercise intensity, measured by analysis of respiratory quotient [15]. The reintroduction of the needle muscle biopsy technique in the late 1960s [12,30,31] provided direct measurements of intramuscular substrate utilisation. Subsequent non-invasive methods such as magnetic resonance spectroscopy [32] and contemporary isotope methodologies [29] also advance our understanding of muscle metabolism. Current advances in molecular biology and its techniques have also increased knowledge in the molecular mechanisms underpinning structure, function and regulation of muscle metabolism and the adaptive response to exercise training [33]. The culmination of human metabolism assessment techniques allowed for a greater knowledge of the relative contribution of these endogenous stores to energy provision and the several factors that may influence metabolism, such as diet, sex, fibre types, environment, training status and the duration, intensity and mode of exercise [28,29,34,35,36].While the exploration of substrate metabolism methods will not be covered in this review, the reader is referred to the following resources on this topic [33,37,38,39].

In exercise physiology, exercise can be broadly characterized as either low load, high repetition (endurance activities) or high load, low repetition (weight or resistance training) [40,41], and both exercise modalities are commonly incorporated into training programs at various times or stages [42]. Across this strength–endurance continuum is a broad range of intermittent exercise activities (i.e., exercise that involves alternating between two different intensities such as football and basketball) with a mixture of fuel-generating sources dependent on the characteristics of the distinct exercise stimulus [43]. Endurance activities are commonly characterized by an activity duration that exceeds two minutes, including running, cycling and swimming. The capacity to perform endurance-type activities relies primarily on oxidative/aerobic metabolism [29,44]. Certainly, the aerobic energy system is fundamental for humans by serving as a primary source of energy in a broad range of activities, from household chores and walking to marathon running and beyond [27,43,45]. This present review explores substrate metabolism in prolonged endurance-type exercise, defined as “the capacity to sustain a given velocity or power output for the longest possible time” [46]. We also considered regulatory mechanisms ensuring ATP production matches the ATP demand of this type of exercise activity.

## 2. Muscle Glycogen Metabolism during Prolonged Exercise

Endogenous carbohydrates are mostly stored as glycogen in the skeletal muscle and liver [47,48]. Skeletal muscle represents the most abundant glycogen depot due to its much greater mass than the liver [49,50]. In other words, skeletal muscle serves as the predominant disposition (79%) of endogenous carbohydrate stores [51]. Under normal conditions, glycogen content in the muscle ranges between 300–400 mmol glucosyl units·kg dry mass^−1^ [34]. Manipulation through exercise training and diet seems to evoke an increase in muscle glycogen content to about 700–900 mmol glucosyl units·kg dry mass^−1^ [52]. Glycogen stores are therefore finite in nature (<3000 kcal) and hence may limit the capacity for moderate to high intensity exercise (≈50–85% VO_2max_ lasting >45 min, while fat stores are abundant in the body (>100,000 kcal for a 75 kg individual with 15% body fat) and may theoretically support many hours of exercise at the same intensity [30,44,53].

**Figure 1:**
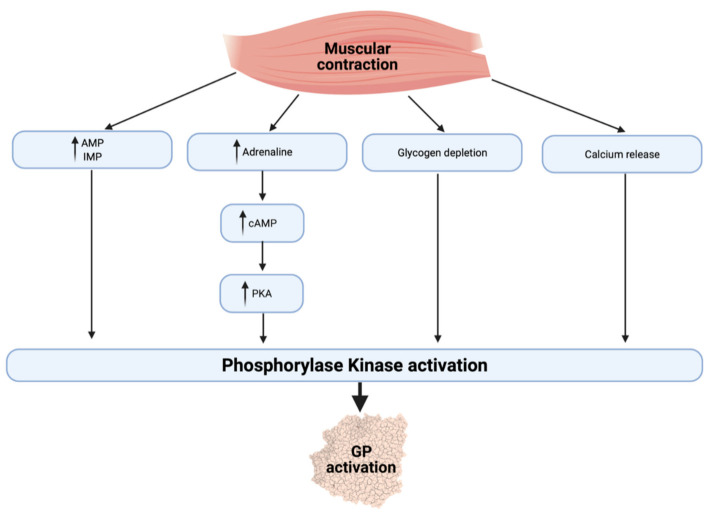
Regulation of glycogen phosphorylase during exercise. AMP, adenosine monophosphate; IMP, inosine monophosphate; cAMP, cyclic adenosine monophosphate; PKA, protein kinase A; PK, phosphorylase kinase; GP, glycogen phosphorylase.

Muscle glycogen degradation depends on the activation of glycogen phosphorylase and debranching enzymes, which ultimately split glucose residues from the glycogen chain [54]. Activating glycogen phosphorylase during exercise depends on a number of factors (Figure 1), allowing for a close regulation of glycogenolysis and oxidation with the different energetic requirements of the working muscle [55]. Glycogen phosphorylase is activated by allosteric binding of adenosine monophosphate (AMP) and inosine monophosphate (IMP) so that the enzyme is responsive to the energy state of the cell. Muscular contraction also increases cytosolic calcium (Ca^2+^) release and an adrenaline-mediated increase in cyclic AMP (cAMP), thereby activating phosphorylase kinase (PK) and the resultant activation of glycogen phosphorylase [56,57].

Muscular contraction also activates AMP-activated protein kinase (AMPK) by an increase in the cellular AMP/ATP ratio when there is a drain on ATP [58]. AMPK is a conserved fuel-gauge and has a key role in intracellular energy homeostasis. Once activated, ATP-utilizing pathways are inhibited and ATP-generating pathways are activated [58]. Hence, AMPK activates glucose uptake, glycolysis and fatty acid oxidation, while inhibiting glycogen and protein synthesis. It appears that the degree of AMPK activation depends on the level of metabolic stress caused by several factors, including exercise [59]. Prolonged AMPK activation can lead to a chronic adaptation to endurance exercise, like mitochondrial biogenesis and increased GLUT4 expression [60]. AMPK is essential for endurance exercise [61]. It has been shown that lacking AMPK in mice skeletal muscle led to a notably impaired ability to perform muscle contractions and reduced voluntary wheel running [62].

Calmodulin, the δ-subunit of phosphorylase kinase, associates with the protein troponin that stimulates skeletal muscle contraction [63]. It is a regulatory protein that acts as a calcium sensor and translates the Ca^2+^ signal into a cellular process [64]. An increase in Ca^2+^ concentration in the cells, via activation of phosphorylase kinase, triggers glycogen degradation [65,66].

An acute exercise bout’s intensity and duration predominantly influences muscle glycogen degradation rates. As a consequence, muscle glycogen degradation exponentially increases with exercise intensity and, over time, progressively declines due to the finite nature of this carbohydrate store within skeletal muscles [67]. Another important factor for glycogen metabolism is the availability of this substrate prior to exercise [68], with elevations in the rate of degradation of glycogen during exercise being exponentially related to pre-exercise muscle glycogen concentrations [69,70]. In support of this, increasing the glycogen content in one leg was shown to accelerate glycogenolysis twofold compared with the control leg with low glycogen content, which is likely ascribed to a greater increase in the activity of glycogen phosphorylase concomitant to its greater binding to the glycogen granule [71]. This demonstrates that glycogen availability prior to exercise may modulate its metabolism during subsequent exercise [72].

The mode of exercise and whether or not carbohydrates were ingested during exercise seem to be interrelated determinants of equal importance on utilising muscle glycogen during physical work [73]. For example, when ingesting carbohydrates during constant pace running, carbohydrate oxidation can be maintained, thus sparing muscle glycogen through preferential oxidation from this exogenous carbohydrate source [25], in particular type I muscle fibres [74]. In contrast, the majority of cycling-based investigations suggest that this phenomenon is not likely to provide an ergogenic benefit when carbohydrates are ingested [73], albeit glycogen sparing was also observed during the first hour of cycling when fed carbohydrates [75]. Rather, an enhanced maintenance of euglycemia towards the end of exercise [76] and a suppression of endogenous glucose production [77] are likely mechanisms by which exogenous carbohydrate intake improves cycling performance during prolonged exercise. Furthermore, glucose infusion was shown to improve cycling endurance capacity independent of total carbohydrate oxidation or hypoglycaemia, suggesting that maintenance of glycaemia when glycogen stores are low may have an ergogenic effect [78], possibly via a central mechanism whereby compromised bodily carbohydrate availability limits cerebral glucose uptake, thus amplifying perceived effort and reducing motor activation [79,80]. It can therefore be concluded that the ingestion of carbohydrates presents an ergogenic effect during prolonged moderate to high-intensity endurance exercise, and the physiological mechanisms responsible for these observations appear to involve several interrelated factors including: maintenance of euglycemia and an attenuation of central nervous system fatigue, glycogen sparing, and reduced exercise-induced strain [81,82].

Sex differences may play a role in the discrepancies in the utilisation of endogenous carbohydrate stores [35,83]. It has been reported that increasing carbohydrate intake increases glycogen storage to a lesser extent in women when compared with men [84], albeit these observations are likely to be attributable to the difference in total energy intake [85]. Evidence indicated no sex differences in the ability to synthesise glycogen [86]. Additionally, men seem to have lower rates of fat oxidation at submaximal exercise and an earlier shift to using carbohydrates as the dominant fuel when compared with women [87]. More recent evidence demonstrates that women tend to have lower resting muscle glycogen concentrations, and consequently a decline in net glycogen utilization under these circumstances [71] would be expected to favour a shift toward fat oxidation [44].

Training status affects the selection of substrate use during exercise as a result of an enhancement in lipid oxidation and a concurrent improved insulin-stimulated glucose uptake capability when compared with untrained individuals [88,89,90]. Endurance training influences substrate selection during exercise and thus contributes to the rate of endogenous carbohydrate metabolism during exercise [21,91,92,93]. Nevertheless, it should be noted that large inter-individual differences exist in the capacity to oxidise lipids [38]. In a cross-sectional study, Venables et al. (2005) demonstrated that lean body mass, fat mass, sex, VO_2max_ and estimated physical activity level can only account for 34% in the variability in peak fat oxidation and thus the inter-individual variation in fat oxidation remains largely unexplained [87]. However, it is likely that a degree of this variation can be accounted for by diet, given that altering the diet to a high carbohydrate/low fat or high fat/low carbohydrate diet can substantially supress or elevate fat oxidation, respectively [94]. Other factors may impact the degradation of muscle glycogen, such as dehydration and hyperthermia [36,95,96,97].

## 3. Extra-Muscular Carbohydrate Metabolism during Prolonged Exercise

Although muscle glycogen plays a central role in energy metabolism during moderate to high intensity exercise, the importance of other extra-muscular carbohydrate sources (e.g., liver glycogen and lactate) is profound when performing prolonged exercise [26]. The remarkable contribution of these carbohydrate sources with prolonged duration is seen even at low (≈30% VO_2max_) exercise intensities, whereby splanchnic glucose production was sufficient to deplete 75% of the liver glycogen stores [98]. These two sources are by no means exclusive of each other, and the fact that increased glucose uptake by the working muscle is quantitatively matched by the liver seems to support this notion [99]. The liver has a central role in blood glucose homeostasis [100], which is in turn critical for supplying glucose to the various tissues in the post-absorptive period [101] and physical exertion [102]. The importance of the liver during exercise is also underscored by being the only organ that is capable of considerable glucose production and assimilation [103] and a substantial disposal of the end products of muscle metabolism [99].

In healthy individuals, the liver ensures the maintenance of glycaemia within a tight range under widely divergent physiological conditions. This is achieved through the dynamic equilibrium of hepatic glucose production mechanisms (namely glycogenolysis and gluconeogenesis) by which the relative contribution is determined by the intensity and duration of exercise, in addition to the absorptive state of subjects [99,104]. Under a resting postprandial state, the production of blood glucose relies primarily on glycogenolysis [105]. Nevertheless, the rapid utilisation of hepatic glycogen and the relatively small quantities of this substrate evoke a gradual decline in these stores [106]. This rapid depletion of hepatic glycogen emphasises the importance of gluconeogenesis with duration [106], as was shown with a 54% contribution to glucose production via gluconeogenesis following a 14 h fast [107]. Thus, recent estimations for the contribution of liver glycogenolysis and gluconeogenesis in glucose turnover are approximately equal (50%) in healthy post-absorptive humans [106]. The regulation of hepatic glucose production occurs primarily through circulating insulin levels, despite the fact that basal glucagon levels are required for the stimulation of glucose output in the overnight fasted state [108]. This is evidenced by the modulating influence of insulin on hepatic glycogen degradation in both canines [109] and humans [110], although the sensitivity of glycogenolysis and gluconeogenesis to insulin concentration may differ substantially [110]. In addition, other indirect insulin-related mechanisms were also observed, such that reductions in lipolysis, muscle proteolysis and glucagon secondary to insulin activity cannot be overlooked [111].

Similarly, during exercise, the contribution of both glycogenolysis and gluconeogenesis cannot be overstated in the absence of nutrient (carbohydrate) ingestion. In fact, an appreciable amount (15–30%) of the energy required for moderate exercise is obtained from blood glucose [101], while supplying most of the fuel for the central nervous system (CNS) [102]. However, the matching of glucose disposal by the muscle and the appearance of glucose into the circulation during exercise presents a major challenge for this organ [102,112]. Under postprandial conditions, these complexities during prolonged exercise may be related to the inability of liver glucose output to match the required muscle glucose uptake [51]. This would appear to be an inevitable outcome since the energy requirements for a given exercise intensity remains constant, whereas liver glycogen stores are limited [99]. During moderate-intensity exercise, the hepatic glucose production was shown to increase threefold from resting conditions [113], which was suggested to be in response to the exercise-induced increments in glucose uptake [102]. This suggests that hepatic glucose output is the primary extra-muscular source of glucose for the working muscle [106]. It should be noted that although the kidney is capable of producing glucose, its contribution to glucose output during exercise is negligible [101].

Glucose output is almost entirely derived from liver glycogenolysis at the initial stages of prolonged moderate to high-intensity exercise secondary to glucagon and noradrenaline-mediated activation of hepatic glycogen phosphorylase [101,102]. Indeed, hepatic glycogenolysis is directly related to hepatic glycogen content and thus supports the notion that glucose output is primarily dependent on liver glycogen content [114]. Gluconeogenesis also plays an essential role in maintaining total glucose production during moderate to high-intensity exercise. Gluconeogenesis was shown to contribute to ≈20% of total glucose production during exercise at ≈70% VO_2max_, [115]. The latter study employed tracer methodology to measure glucose flux, which in contrast to venous blood sampling allows accurate partitioning of total glucose production.

Glucoregulation is accomplished by a combination of regulatory controls, namely through feedback [116] and feedforward [117] mechanisms. The concentration of blood glucose plays a central regulatory function, which closely monitors the demands for substrate mobilisation [102]. In accordance, it was shown that exogenous carbohydrate ingestion mediates a blunting of liver glucose output during exercise [77]. Moreover, it was observed that patients with McArdle’s disease demonstrate a more pronounced mobilisation of extra-muscular carbohydrate sources during exercise when compared with healthy controls, to compensate for the lack of intramuscular glycogenolysis [118]. Taken together, blood-borne and neural feedback mechanisms appear to exert an important role in the modulation of exercise-induced hepatic glucose output [100]. With regards to feedforward mechanisms, it was reported that blood glucose concentrations exhibit a disproportionate increase at the onset of exercise as a result of a rise in blood glucose production from the liver that relatively exceeds glucose uptake [117]. This seems to support a contribution from CNS to regulate blood glucose concentration and thus indicate a feedforward governance of glycaemia [102]. It is understood, nonetheless, that these mechanisms of glucoregulation coexist and that decipherment between them is intricate, given that the experimental isolation of one pathway is likely to be compensated by the other [102].

**Figure 2:**
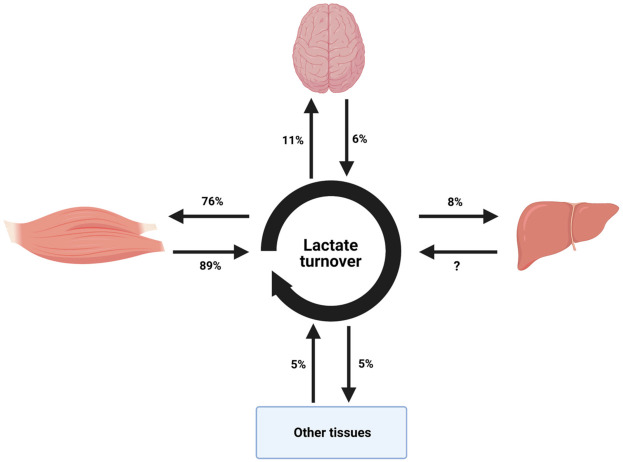
Diagram representing tissue contributions to systemic lactate turnover during moderate exercise in healthy people. This diagram only provides an estimate as a result of differences in exercise intensity, durations and tracer use between studies amalgamated to reflect lactate turnover during exercise. Reproduced with permission from van Hall, Acta Physiologica; published by John Wiley and Sons, 2010.

Lactate is a crucial intermediate in energy metabolism and it is known to be produced in many tissues including the skeletal muscles and liver (Figure 2). Lactate produced in the skeletal muscles is transferred through the blood stream to the liver where it undergoes gluconeogenesis to produce glucose [119]. This produced glucose re-enters the blood stream and then the skeletal muscles where it can be metabolized again to lactate by glycolysis (Cori cycle) [119]. Blood lactate oxidation contributes to about 30% of overall carbohydrate metabolism during moderate to high-intensity (70–75% VO_2max_) endurance exercise [120]. Most lactate disposal occurs via oxidation in working skeletal muscle during prolonged endurance exercise, but other tissues such as the brain, liver and kidneys are also involved in lactate turnover [121,122,123]. However, as a glycolytic end product in muscle, lactate is an important precursor for hepatic gluconeogenesis and the production of glucose during exercise, which may subsequently undergo oxidation [98,124]. This is even important for glycogen storage and degradation [125,126]. Recent stable isotope tracer methods report that 90% of lactate was directly oxidised during prolonged endurance exercise at ≈70% VO_2max_, while 10% of lactate was indirectly oxidised via gluconeogenesis [120]. The metabolic activity of blood lactate coupled with that of hepatic glycogenolysis and gluconeogenesis is likely to contribute substantially to energy provision during prolonged moderate to high intensity exercise [115,120]. During exercise, glycolytic flux in the skeletal muscles can be dramatically increased [127,128]. In order to allow this increase in flux, the enzymes of this pathway must be activated. The key regulatory enzyme for glycolysis is phosphofructokinase (PFK). PFK has been demonstrated to be upregulated in times of metabolic demand [129]. An accumulation of citrate, a negative PFK modulator, inhibits glycolysis pathway by regulating PFK. On the other hand, AMP or ADP are accumulated when ATP is depleted; signals that ATP is required result in an active glycolytic pathway [27].

## 4. Lipid Metabolism during Prolonged Exercise

Fat oxidation is the second dominant substrate for endurance exercise [51,130]. Although carbohydrates are a preferred substrate during moderate to high-intensity exercise and are likely to determine exercise capacity during such endurance activities [26,131], sustained endurance capacity and the attenuation of glycogen depletion are largely mediated by the metabolism of fat by providing fatty acids (FA) for β-oxidation in the mitochondria [132,133,134]. The utilisation of fat during high energy demand (i.e., exercise) would appear to be imperative; fat is the most abundant endogenous energy depot that is >60 times the amount stored as glycogen (<3000 kcal) in humans, although this is heavily dependent on adiposity [134], whereas carbohydrate availability is limited and therefore is causally related to limitations in the capacity for exercise [135]. During endurance exercise, FA oxidation permits sustained exercise and delays the onset of glycogen depletion and hypoglycaemia. [136]. An intricate interaction exists between carbohydrate and fat metabolism, a number of regulatory mechanisms have been established to explain the interplay between these two main substrates (for a review see [137]). In concurrence, it is apparent that factors such as intensity and duration of exercise [138], training status [139], diet [135] and sex [140] have a profound effect on the utilisation of substrates during exercise.

The use of fat as an energy substrate involves hydrolysis of triacylglycerols (TG) to FA and glycerol (i.e., lipolysis), and the delivery of the released FA for oxidation in skeletal muscle mitochondria. TG can be derived from three sources: adipose tissue TG, intra-myocellular TG (IMTG) and circulating plasma TG [141]. During exercise, the contribution from circulating plasma TG is minimal [142]. Despite the relatively small quantities of IMTG compared with adipose tissue TG, recent understanding suggests that IMTG represent a considerable portion of the fat used during endurance exercise [143,144]. The use of IMTG during exercise, calculated indirectly with isotopic tracer methods, has been estimated to provide more than 50% of the total fat oxidized [145]. This may be related to its proximity to the mitochondria [143,144] and its two to threefold greater availability in type I muscle fibres that exhibit greater fat oxidation capabilities [146]. Nonetheless, controversy still exists on the role of IMTG as a source for β-oxidation that mainly relate to methodological complexities to accurately quantify the contribution of this substrate and the consequent inconstancies of its role during prolonged exercise in the literature [146]. With this in mind, a consistent finding was that non-TG fat sources contribute significantly to moderate intensity exercise [28,29,143].

TG may practically serve as an unlimited energy source to support the energetic requirements during endurance exercise, given their quantitative superiority to be stored over carbohydrates, and their higher energy density than carbohydrates [130]. On the other hand, numerous factors can limit the reliance on fat as an energy source during prolonged endurance-type exercise. In contrast to carbohydrate oxidation, whereby the glucose production and uptake by the active muscles are sustained provided sufficient glycogen availability [102], there is no evidence of such a mechanism to control the availability–utilisation cycle of FA to the energetic requirements of the working muscle [147]. An 8–10-fold increase (above resting amounts) in whole-body fat oxidation is observed during low to moderate-intensity exercise, largely derived from TG as a result of its increased availability in circulation, uptake and oxidation by the exercising muscle secondary to β-adrenergic stimulation [28,29]. While this enhancement in fat oxidation is presumably linked to a substantial increase in lipolysis and adipose tissue perfusion via increased blood flow [130,148], this relationship does not hold in higher intensities [133]. Fat oxidation during high-intensity exercise is inhibited to values below those noted in low-intensity exercise. At moderate to high-intensity exercise (≈75% VO_2max_) the contribution of fat oxidation is blunted by ≈34% compared with fat utilisation rates during lower intensities (≈55% VO_2max_) [29]. In a similar manner, the oxidation of fat at higher intensities (≈85% VO_2max_) becomes down-regulated compared with moderate intensities (≈65% VO_2max_) [149]. The suppression of fat use during high-intensity exercise appears to dissociate from a decline in plasma non-esterified free fatty acids (NEFA) availability and/or blood flow [29].

A number of factors have been suggested for the down-regulation of fat oxidation at higher exercise intensities. Failure in adipose tissue to supply the exercising muscle with sufficient FA may be related to an inhibition of fat mobilisation or an inadequate perfusion of the adipose tissue [133]. Secretion of hormones related to energy substrate oxidation are affected by exercise intensity [150]. For example, secretion of catecholamines increases exponentially with exercise intensity [151], which mediates an α-adrenergic inhibition of lipolysis and thus antagonizes β-adrenergic stimulation of lipolysis [152]. Other hormonal stimuli of lipolysis such as insulin, growth hormone and cortisol may also be involved [133]. Moreover, using positron emission tomography (PET), it was recently demonstrated that the infusion of noradrenaline (an α-adrenoreceptor agonist) reduced adipose tissue blood flow by ≈40% both at rest during low-intensity single leg exercise [153]. Thus, it is possible that a reduction in adipose tissue blood flow secondary to the elevations in catecholaminergic response with increased exercise intensity may also be involved in the limitations in fatty acid delivery. The concentration of lactate is also increased with exercise intensity [29], coupled with the knowledge that the lactate receptor G-protein-coupled receptor (GBR81) is expressed in adipose tissue, and that this receptor mediates a marked anti-lipolytic action [154].

When focussing on the limitations of FA oxidation from the transition from moderate to high-intensity exercise, the failure of the muscle to oxidise FA in the mitochondria may also be a candidate. The oxidation of long chain FA (i.e., FA with >12 carbon atoms) have to be converted to their acylcarnitine form to enter the mitochondria for β-oxidation by a reaction catalysed by carnitine palmitoyltransferase 1 (CPT-1) [142]. Indeed, increasing exercise intensity was shown to result in a decrease in free carnitine pool [155] and CPT-1 activity, dependent on the availability of carnitine [156]. Thus, the reduction in intra-muscular free carnitine limits the ability of CPT-1 to transport long chain FA to the mitochondria [157]. Therefore, it is possible the carnitine is a major regulator of FA oxidation with increasing exercise intensity [142].

Malonyl-CoA is an intermediate in the de novo synthesis of FA and is an allosteric inhibitor of CPT-1, thereby inhibiting fat oxidation [158]. It is formed from carboxylation of acetyl CoA through the action of acetyl-CoA carboxylase (ACC), the key regulatory enzymes of FA synthesis [159]. Increases in FA uptake into skeletal muscle with exercise is accompanied by a reduction in ACC activity [141]. In summary, an important adaptation to endurance training is a shift in proportionate substrate utilisation from carbohydrate towards fat oxidation, mainly to preserve the limited endogenous carbohydrate stores [136]. This is principally achieved by an augmentation in mitochondrial volume, FA transport and enzymatic adaptations (e.g., increase hydroacyl-CoA-dehydrogenase) to use fat, and a reduction of cell signals such as ADP and AMP during exercise at submaximal work rates thus reducing the activation of key enzymes (e.g., glycogen phosphorylase and pyruvate dehydrogenase) of carbohydrate metabolism [92,160]. Furthermore, it has been demonstrated that nutritional manipulation (i.e., high-fat diets) may provide an additive but distinct adaptation to augment the rates of fat oxidation [135], although the efficacy of such practices on endurance capacity are equivocal [161] and may have health risk implications [162]. The intensity and duration of the exercise bout appear to be a crucial factor in selective substrate utilisation [28,29]. It has been shown that the maximal rate of fat oxidation occurs at intensities of ≈65% VO_2max_ [163], with greater oxidation rates in running than in cycling [164], although unexplained individual variation to oxidise fat also exist irrespective of training status and sex [87]. At higher intensities than those suggested to maximise fat oxidation rates, oxidation of fat declines markedly, possibly by the down-regulation of lipolysis via lactate, catecholamine-mediated stimulation of α-adrenergic receptors, and consequently a reduction in adipose tissue blood flow and/or a reduction in the muscle’s ability to oxidise FA in the mitochondria secondary to reductions intramuscular free carnitine [133,142].

## 5. Protein Metabolism during Prolonged Exercise

Protein metabolism serves as an auxiliary fuel source during prolonged endurance exercise by contributing to ≤5% of ATP provision [165]. Although some estimates postulate that higher percentages of energy metabolism are derived from intra- and extra-muscular protein sources that may reach 20%, the contribution of amino acids is clearly relatively small in comparison to the predominant fuel sources (i.e., carbohydrates and fat) [166]. From a quantitative standpoint, protein is less important than other substrates to supply energy during prolonged exercise [167], but by no means is the contribution from protein negligible, as evidenced by the profound alterations in whole body protein metabolism and amino acid kinetics in response to exercise that consequently regulate fuel metabolism and the adaptive response to training [166,168,169,170].

During exercise, the branched-chain amino acids (BCAA) are preferentially oxidised to other forms of amino acids through a transamination process to become transferred into keto-acids by the rate-limiting enzyme branched-chain oxo-acid dehydrogenase (BCOAD) [171,172]. The activity of BCOAD was shown to be elevated from the transition from rest to exercise, implying an analogous increase in BCAA oxidation by the working skeletal muscle [168,173]. Studies have corroborated these findings by demonstrating an increase in leucine oxidation during endurance exercise [174,175]. The disruption of BCAA metabolism was shown to severely impair endurance exercise to exhaustion within the skeletal muscle of rodents [176]. It seems paradoxical therefore that endurance training results in a reduction in leucine oxidation [177]. Indeed, it was observed that following endurance training (90 min of 65% VO_2max_) leucine oxidation and BCOAD activation were lower in both males and females [171]. However, an examination of leucine kinetics and BCOAD activation revealed that BCOAD capacity was higher following training, indicative that the absolute capacity for BCAA oxidation had increased and a concomitant exercise-induced efficiency in protein metabolism was observed [165,171]. These findings should be interpreted with caution, as other investigations showed below baseline leucine oxidation levels during 3 h of cycling at 75% VO_2max_ [178] and that the proportion of protein utilisation during exercise remains largely equivocal, possibly due to methodological limitations [179].

The role of protein metabolism during exercise has also been linked to the provision of precursors for tricarboxylic acid (TCA) reaction; a major common pathway for the oxidation of carbohydrates, fats and amino acids [180,181]. The oxidation of BCAA may induce a cataplerotic state on TCA cycle intermediates, as the catabolism of BCAA requires the consumption of a specific intermediate from this cycle (2-oxoglutarate), which theoretically could impair TCA cycle flux and hence aerobic energy provision [168,182]. Data from animal studies appear to support the notion that aerobic energy delivery is unaffected by TCA cycle reductions [183]. In humans, a dissociation between TCA cycle pool size and aerobic phosphorylation was demonstrated [184,185], suggesting that TCA intermediate content does not represent any functional importance to oxidative phosphorylation [182].

Alanine is a dispensable amino acid that can be synthesized endogenously by the liver, acting as an subsidiary source of energy during extreme circumstances such as starvation and prolonged endurance exercise [186]. This process is mediated via a distinct metabolic pathway known as the glucose alanine cycle proposed in the late 70s by Philip Felig and colleagues [98]. During this process, alanine is shuttled to the liver via the bloodstream and converted into pyruvate through a transamination reaction catalysed by the action of glutamate-pyruvate aminotransferase. Pyruvate can then act as metabolic substrate via a gluconeogenic pathway, where the newly formed glucose can boost exercising muscles for their energy demands. Accordingly, it seems that gluconeogenesis tends to be the predominant source of energy during the late phase of prolonged exercise. In this regard, a series of experimental metabolic studies on animal models by Wasserman and colleagues (1988) demonstrated that the relative contribution of gluconeogenesis via glucose-alanine cycle to the total hepatic glucose production was approximately 15% during the first one hour of exercise. However, it reached 20% to 25% when exercise continued for another one hour and a half [187].

## 6. Other Energy Provision Sources

### 6.1. Glycerol

Glycerol is a hydrophilic carbon skeleton of sugar alcohol that can be easily transported as a free molecule in the bloodstream in response to triglyceride hydrolysis and FA mobilization. Lipolytic activity within adipose tissue and intramuscular fat is highly active during moderate-intensity endurance exercise by the action of catecholamines, leading to elevated circulating glycerol levels [134,188]. Glycerol cannot be metabolized by skeletal muscles (as they lack glycerol kinase enzyme). However, glycerol is shuttled to the liver via the blood where it is phosphorylated by glycerol kinase providing a precursor for gluconeogenesis to assist the liver in maintaining glucose output as glycogen levels decline [189].

### 6.2. Ketones

Ketone bodies are lipid-derived low molecular weight hydrophilic molecules, and are normally found in circulation in relatively small quantities ranging between 0.1 to 0.5 mmol/L [190]. They are synthesised in the mitochondria by the liver through a metabolic process known as ketogenesis [191]. The central precursor for the ketogenic process is acetyl CoA, derived predominantly from excessive oxidation of fatty acid, which in turn is converted into acetoacetate, β hydroxy butyrate and acetone. In order to be utilized as a metabolic substrate for energy expenditure, ketone bodies are excreted by the liver to the bloodstream and reconverted into acetyl-CoA in extrahepatic organs, and thereafter are incorporated into the TCA cycle for terminal oxidation [192]. Therefore, ketone bodies are widely recognized as an alternative source of energy for highly aerobic metabolic organs such as the brain, skeletal muscle, and heart, particularly when carbohydrate availability is deficient or during periods of negative energy balance accounting for approximability 185 g/day as seen in fasting and starvation states, low carbohydrate high fat ketogenic diets or during prolonged endurance exercise [191,193,194]. The metabolic contribution of ketone bodies as a potent metabolic fuel for energy expenditure during exercise and physical activity is an area of increasing attention in the field of exercise physiology and sport nutrition in recent years. However, recent evidence questions the potential ergogenic benefits of low carbohydrate ketogenic diets and exogenous ketone supplementation on endurance-type exercise [193,195], and the potential negative health consequences [196].

## 7. Conclusions

**Figure 3:**
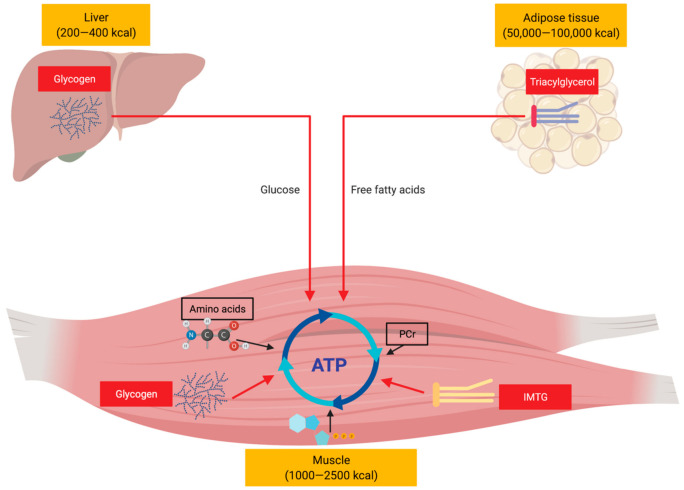
Simplified overview illustrating the major fuel sources supporting endurance-type exercise. ATP, adenosine triphosphate; PCr, phosphocreatine; IMTG, intramyocellular triacylglycerol.

After over a century of research, the regulation of metabolism in endurance-type activities remains an area of interest for exercise physiologists, biologists and nutritionists [10,160,197,198,199]. We have presented an overview (Figure 3) of our current understanding of substrate utilization, metabolic perturbations, regulation and integration in prolonged endurance-type exercise. It is now widely accepted within scientific communities that the three major macronutrients including carbohydrates, fat, and protein are oxidized simultaneously as metabolic substrates for energy expenditure during prolonged endurance exercise, but their relative contributions to supporting the energetic demands of exercise depends on various factors. These factors vary and may encompass environmental, nutritional, physiological and genetics factors. Accordingly, a large number of studies on both human and animal models have been conducted over the last century, aiming at understanding the mechanistic and/or metabolic basis behind substrate utilization during exercise in order to optimize training and performance through nutritional strategies via dietary manipulation of carbohydrate, fats and protein intake.

Endurance exercise improves overall health and enhances sport performance through a shared mechanism of increased metabolic activity in response to a high energetic and/or oxygen demand of the contracting skeletal muscles; as a result, different bodily systems respond in an integrative fashion to meet this homeostatic challenge. If said exercise stimulus is repeated over time, chronic structural and functional changes occur [2,200]. Indeed, while much knowledge has been gained over the past century, much more remains to be explored [10]. For example, the magnitude of exercise-induced utilization of specific storage pools remains to be documented using “real-world” exercise protocols that are relevant to both training and competition scenarios. Whilst the specific regulatory control points of carbohydrate metabolism are now well documented, the precise molecular mechanisms underpinning the regulation of carbohydrate transport, storage and utilization are not yet fully known. Additionally, the identification of the glycogen granule as a regulator of acute exercise response and influence on training adaptation has opened a new field of study that is likely to dominate the applied nature of sport nutrition research in the coming decade. Furthermore, besides exercise induced physiological and metabolic adaptations in parallel with nutritional manipulations, the mechanistic bases whereby regular exercise promotes psychological and mental health is another area of increasing attention in the field. It is now widely thought that exercise induces positive mental health and improved cognitive function via enhancing neuroplasticity and growth factor expression, as well as reducing stress related complications [201]. Therefore, from the early studies from the pioneers in the field to the current state and future perspectives gained, it is clear that exercise metabolism and the bioenergetics field remain as relevant to human health and performance as ever.

## Data Availability

Not applicable.
